# Severe COVID-19 and Sepsis: Immune Pathogenesis and Laboratory Markers

**DOI:** 10.3390/microorganisms9010159

**Published:** 2021-01-12

**Authors:** Mai M. Zafer, Hadir A. El-Mahallawy, Hossam M. Ashour

**Affiliations:** 1 Department of Microbiology and Immunology, Faculty of Pharmacy, Ahram Canadian University (ACU), 6th of October 12566, Egypt; mai_zafer@hotmail.com; 2 Department of Clinical Pathology, National Cancer Institute, Cairo University, Cairo 11796, Egypt; hadir38@hotmail.com; 3 Department of Integrative Biology, College of Arts and Sciences, University of South Florida, St. Petersburg, FL 33701, USA; 4 Department of Microbiology and Immunology, Faculty of Pharmacy, Cairo University, Cairo 11562, Egypt

**Keywords:** COVID-19, sepsis, cytokines, SARS-CoV-2

## Abstract

The ongoing outbreak of the novel coronavirus disease 2019 (COVID-19), induced by severe acute respiratory syndrome coronavirus 2 (SARS-CoV-2), has taken a significant toll on people and countries all over the world. The pathogenesis of COVID-19 has not been completely elucidated yet. This includes the interplay between inflammation and coagulation which needs further investigation. The massive production of proinflammatory cytokines and chemokines results in the so-called cytokine storm, leading to plasma leakage, vascular hyperpermeability, and disseminated vascular coagulation. This is usually accompanied by multiorgan failure. The extensive changes in the serum levels of cytokines are thought to play a crucial role in the COVID-19 pathogenesis. Additionally, the viral load and host inflammation factors are believed to have a significant role in host damage, particularly lung damage, from SARS-CoV-2. Interestingly, patients exhibit quantitative and qualitative differences in their immune responses to the virus, which can impact the clinical manifestation and outcomes of COVID-19. There needs to be a better understanding of the dynamic events that involve immune responses, inflammatory reactions, and viral replication in the context of the COVID-19 infection. Here, we discuss the main aspects of COVID-19 pathogenesis while supporting the hypothesis that inflammatory immune responses are involved in the progression of the disease to a more critical and fatal phase. We also explore the similarities and differences between severe COVID-19 and sepsis. A deeper understanding of the COVID-19 clinical picture as it relates to better-known conditions such as sepsis can provide useful clues for the management, prevention, and therapy of the disease.

## 1. Introduction

Coronavirus disease-2019 (COVID-19) is an infectious disease that has resulted in a catastrophe worldwide. It is caused by severe acute respiratory syndrome coronavirus 2 (SARS-CoV-2), which causes significant infection-related morbidity and mortality. It was first identified in Wuhan, China and has disseminated to other countries causing an ongoing pandemic [1]. Like most infections, the virulence of SARS-CoV-2 occurs on a continuum: some individuals who attain the infection are asymptomatic, others experience mild disease, whereas a small subset of individuals progress to serious or life-threatening COVID-19 [2]. In addition to genetic variation, advanced age, and comorbidities can increase risk and lead to poorer clinical outcomes [3]. This highly-vulnerable subset of patients with COVID-19 has exceptionally alarming laboratory and clinical biomarkers that include very high serum ferritin and D-dimer levels, lymphopenia, hepatic dysfunction, thrombotic tendency, disseminated intravascular coagulation (DIC), and inflammatory bursts that result in multiple-organ failure (MOF) and ultimately death. Additionally, moderate and severe COVID-19 cases present predominantly with respiratory pathology that includes alveolar damage, acute respiratory distress syndrome (ARDS), and reduced oxygen saturation [4]. Severe COVID-19 cases can trigger severe inflammatory responses [4] and patients may end up with septic shock or MOF. The possible causes of this sepsis-like picture might be a dysregulated immune response incapable of controlling the production of excessive amounts of cytokines and chemokines. Alternatively, it could be due to a secondary bacterial infection of the damaged alveoli. Increased cytokine secretion, including IL-2, IL-4, IL-6, IL-10, tumor necrosis factor (TNF)-α, and IFN-γ, in COVID-19 patients has been reported [5]. Genetic and host factors play major roles in viral infections [6]. In this context, the host–pathogen relationship, including correlates of immune dysregulation, such as over-production of pro-inflammatory mediators and pro-inflammatory cytokines that might promote COVID-19 disease progression, must be well characterized [7]. Infection by SARS-CoV-2 involves the action of the virus spike protein (S), which involves angiotensin-converting enzyme 2 (ACE2) as the entry receptor (a metallopeptidase on the membrane of many target host cells) [8], and the engagement of the cellular serine protease TMPRSS2, allowing S2 to facilitate the fusion of the virus envelope with the cell membrane, which facilitates viral RNA entry into the cytoplasm of the target host cells [9]. Although the etiological agent of this highly contagious disease was quickly identified, our knowledge of the virus remains incomplete and inadequate. Further studies are needed to better understand COVID-19 pathogenesis. More data will help avoid misconceptions about the virus and improve prognosis and chances of success in management of the disease. In this review, we focus on COVID-19 pathogenesis and its laboratory markers. We also reflect on the similarities and differences between patients with COVID-19 and patients with sepsis.

## 2. The Dynamic Changes in Cytokine Response

**Figure 1:**
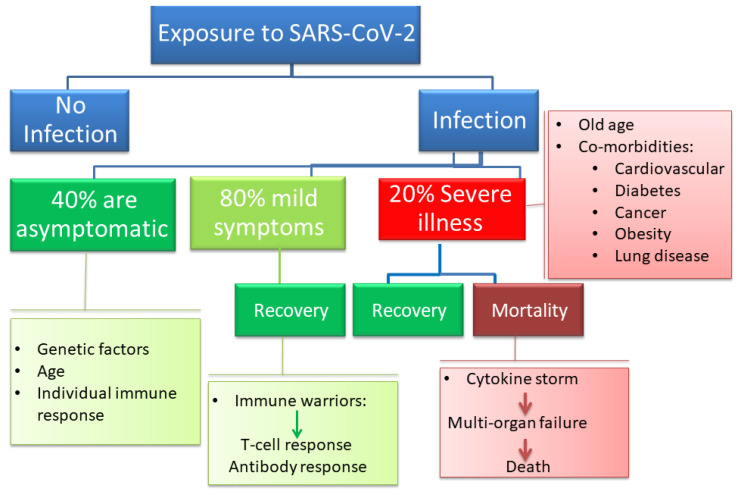
The clinical spectrum of coronavirus disease 2019 (COVID-19) ranges from no or mild symptoms that do not require hospitalizations to severe pneumonia and respiratory failure that can lead to death, especially in aging patients and patients with comorbidities.

Most patients with COVID-19 are not hospitalized as they are either asymptomatic or clinically present with a mild upper respiratory tract-like illness (Figure 1). The clinical symptoms that lead to hospitalization include life-threatening acute respiratory syndrome and pulmonary deterioration, which are the main clinical manifestations of severe COVID-19. This typically happens in patients with high levels of proinflammatory cytokines, in which a cytokine storm is believed to initiate disease pathogenesis [10]. Among the minority of patients that require hospitalization, the percentage of patients that experience cytokine storms is undetermined. Therefore, it is hard to assess the mortality of COVID-19-related cytokine storms. The potent cytokine release mechanisms in response to infections with SARS-CoV-2 appear to be leading to acute lung injury (ALI), ARDS, marked coagulopathy, and MOF in patients with COVID-19, which frequently require intensive-care support [11]. An attempt to correlate serum cytokine concentrations for over 1500 COVID-19 hospitalized patients with disease outcomes revealed that levels of IL-6, IL-8, and TNF correlated with disease outcome and mortality [12]. The cytokine levels were also elevated at the time of hospitalization [12]. The consequences of excessive secretion of IL-6 include activation of the coagulation pathway, increase in vascular permeability, and reduced cardiac function; all these factors contributed to the poor prognosis and disease severity [13]. In one study, the proinflammatory cytokines stayed elevated throughout the disease course except if patients were treated with steroids or remdesivir, which resulted in reduced levels of circulating IL-6 [14]. In another study, T cell numbers in COVID-19 patients were negatively correlated with patient survival and with serum IL-6, IL-10, and TNF-α concentrations [15]. 

## 3. Pathogenesis of COVID-19 in Light of the Damage–Response Framework

The damage–response framework (DRF) is an integrated, flexible, dynamic theory of microbial pathogenesis and infectious diseases that considers the host damage a significant outcome of host–microbe interactions [16]. The DRF provided valuable insights into microbial pathogenesis by shifting the attention from a complete focus on either the host or the microbe to investigating the consequences of microbe–host interaction in the context of infectious diseases. This interaction between the same pair of microbes and hosts can be mutually beneficial at times (commensal relationship) and pathogenic at other times [10].

In the DRF, the host–microbe interaction can be represented by a parabolic relationship between damage in the host and the host’s immune response. In this schema, the host–microbe interaction is represented by a point on the parabola corresponding to a certain amount of damage plotted on the *Y*-axis as a function of the immune response [17]. The position of the patients on the left-hand side of the DRF parabola is indicative that those who have comorbid conditions may have an impaired host’s immune response and thus damage can be caused in the host. The right-hand side of the parabola is indicative that some infectious diseases can be the product of aggressive immune responses [18]. The microbe–host relationship includes correlates of immune protection, such as virus-specific antibodies that control disease [19] and correlates of immune dysregulation, such as proinflammatory cytokine overexpression that may promote disease [20]. Considering different correlates provides a roadmap into the understanding of COVID-19 pathogenesis and clinical manifestations. The initial syndrome of SARS-CoV-2 is characterized by fever and respiratory symptoms. This indicates an immune response that is trying to control the viral infection. Asymptomatic transmission may indicate low viral loads or immune responses that do not provoke clinical signs, symptoms, or damage to the host. Asymptomatic carriers can still disseminate the virus, leading to infections in more susceptible people who will eventually develop clinical disease symptoms [21,22]. Thus, they can contribute to silent epidemics, while at the same time contributing to the establishment of herd immunity [21,22].

Aging is usually accompanied with weaker immunity [23]. COVID-19 occurrence in the elderly population and/or patients who already have numerous comorbidities can result in very high risks of severe disease and death [10]. The immune response of patients having comorbidities might be weaker and less effective in containing the virus. Respiratory diseases and cardiovascular diseases, hypertension, diabetes, and diseases affecting the immune system are associated with significant morbidity and mortality [24]. As COVID-19 progresses, it can lead to hyper-inflammatory responses and cytokine storms [25]. Given that IL-6 is one of the key cytokines that are correlated with disease progression and severity, IL-6 inhibitors may be of benefit to patients with severe COVID-19 [26]. In this context, immunosuppressive drugs might be valuable. Conversely, a recent study suggested that an IL-6 receptor blockade may not be a successful treatment strategy in patients who are moderately or severely ill with COVID-19, as it did not prevent the most severe disease consequences [27]. The discrepancy might be due to individual differences in host immune responses to the COVID-19 infection. Hence, more studies on different patient groups can better clarify the exact role of elevated IL-6 serum concentration in disease progression. In patients with COVID-19 with elevated inflammatory cytokines, post-mortem pathology reports have shown tissue necrosis and interstitial macrophage and monocyte infiltrations in the lung, heart, and gastrointestinal mucosa [28,29]. In addition, severe lymphopenia with functionally exhausted T cells [15] and dysregulated T cells responses [30] are frequently seen in severe cases of COVID-19. Although the T cell activation pattern has emerged as a hallmark of acute COVID-19 in early stages [31,32], several studies reported dysregulated T cell activation in critically ill patients and ultimately T cell exhaustion [33]. Taken together, results pose hard questions about the state of exhaustion of T cells in patients with severe hyperinflammation and its relation to the increased T-cell activation in earlier stages of COVID-19 immune responses.

## 4. Cytokine Storm Syndrome

**Figure 2:**
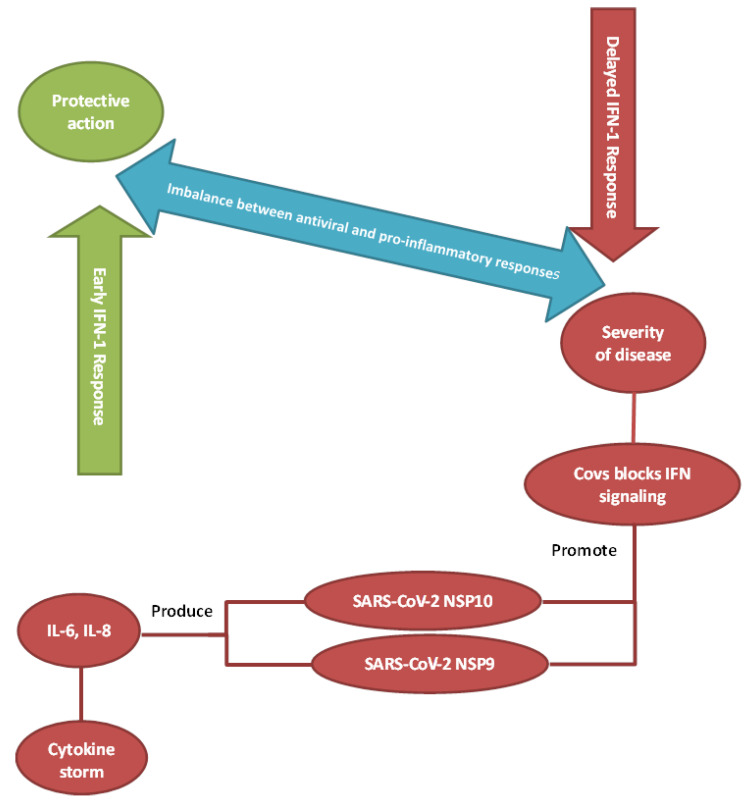
Imbalance between antiviral and proinflammatory responses. Insufficient or inappropriately-timed activation of interferon signaling may play a significant role in disease progression to severe COVID-19.

Cytokine storm syndrome (CSS), cytokine release syndrome, or hypercytokinemia is a cytokine-mediated systemic inflammatory response characterized by an overwhelming release of proinflammatory mediators provoked by a variety of conditions that have different etiologies and outcomes [34]. The host’s immune response to any infection is not only a simple, quick process but is the outcome of complex steps that evolve with time and space and include different cell types [35]. The production of inflammatory cytokines can trigger new cytokine release, which can consequently cause organ damage during severe COVID-19 infections. It appears that when a certain response threshold is eventually reached, sepsis (severe clinical syndrome) can be detected. This can highly influence the morbidity and mortality rates [35]. The clinical consequences of CSS include a presentation of persistent fever, lymphadenopathy, cytopenia, elevated triglycerides and ferritin with accompanying hepatosplenomegaly, progressive organ failure, and eventually death if the high cytokine levels persist and remain uncontrolled over time [36]. From this perspective, respiratory failure is the most noticeable symptom, but the heart and central nervous system are also impacted [37]. In the context of the process, a wide range of proinflammatory cytokines are secreted in an unrestrained way from both arms of the immune system. These include interferon (IFN)-γ, TNF, IL-1, IL-6, and IL-18, and they directly lead to the formation of the dreaded cytokine storm [38] (Figure 2).

Accumulating evidence suggests that cytokine storms are salient features of the most severe cases of COVID-19. In the context of COVID-19, high concentrations of peripheral blood immune mediators have been detected including monokine induced by gamma interferon (MIG), IL-6, IL-9, IL-1β, macrophage inflammatory protein 2-alpha (MIP2-α), TNF-α, IL-2, interleukin-1 receptor antagonists (IL-1RA), IL-7, macrophage inflammatory protein 1-alpha (MIP1-α), MIP1-β, interferon gamma-induced protein 10 (IP-10), IL-8, basic fibroblast growth factor (bFGF), monocyte chemoattractant protein-1 (MCP-1), granulocyte-colony stimulating factor (G-CSF/GCSF), IFN-γ, granulocyte-macrophage colony-stimulating factor (GM-CSF), platelet-derived growth factor (PDGF), IL-10, and vascular endothelial growth factor (VEGF). IL-2, IL-6, IL-7, IP-10, IL-2R, IL-10, TNF-α, MIP1-α, MCP-1, and GSCF levels are positively correlated with disease severity [7].

## 5. Clinical and Laboratory Biomarkers in COVID-19 and Sepsis 

Several abnormalities in patients with COVID-19 are observed using routine laboratory investigations. A meta-analysis of 19 observational studies involving almost 3000 patients with confirmed COVID-19 showed that the most common laboratory findings reported were decreased serum albumin (76% prevalence), increased C-reactive protein (CRP) (58%), increased lactate dehydrogenase (LDH) (57%), lymphopenia (43%), and elevated erythrocyte sedimentation rate (ESR) (42%) [39]. Similar abnormalities in the laboratory findings of patients with COVID-19 were reported in a review of eight smaller studies: lymphopenia (35–75%); increased CRP (75–93%), LDH (27–92%), and ESR (up to 85% of cases); low concentrations of serum albumin (50–98%); elevated D-dimer (36–43%); and low hemoglobin (41–50%) [40]. In-hospital deaths were significantly associated with elevated CRP, advanced age, and impaired renal function [41]. A recent study showed that reduced serum albumin levels were associated with increased mortality in COVID-19 patients [42]. Other laboratory parameters reported in COVID-19 patients were increased neutropenia, total bilirubin, creatinine, cardiac troponin, prothrombin time (PT), and procalcitonin (PCT) [43].

Baseline serum ferritin level (≥ 500 ng/mL) is a prognostic marker of severe and lethal COVID-19 and an independent risk factor for disease severity and bilateral lung infiltrations [44]. Ferritin is a major mediator of immune dysregulation, particularly in cases of excessive hyperferritinemia through pro-inflammatory effects contributing to the cytokine storm [45]. Hyperferritinemia has shown a positive correlation with levels of CRP (*p* < 0.0001) and a negative correlation with lymphocyte counts (*p* < 0.03). High serum ferritin levels are significantly higher in non-survivors than in survivors [45]. An analysis involving 69 patients critically-ill with COVID-19 showed elevated levels of ferritin compared with patients with mild disease [45]. In line with this, high serum ferritin levels were detected upon hospital admission and throughout the hospital stay in patients who died by COVID-19 [46]. It is believed that there was a non-stop increase in serum ferritin levels in these hospitalized patients to the extent that the median values of serum ferritin levels after day 16 of hospitalization exceeded the upper limit of detection of ferritin levels in these patients [46]. As such, elevated serum ferritin is thought to be a crucial factor influencing the severity of COVID-19 [47]. The association of elevated ferritin and COVID-19 disease severity may be related to the cytokine storm or may be due to tissue damage that is similar to the damage that occurs due to hyperferritinemia in severe sepsis and septic shock. Importantly, ferritin is elevated in both sepsis and COVID-19. Thus, hyperferritinemia can be a marker of uncontrolled inflammation. It has been proposed that markedly elevated ferritin is a key mediator of immune dysregulation through proinflammatory and immunoregulatory activities [48]. Cytokine storms that are induced in patients critically-ill with COVID-19 can trigger the coagulation cascade, resulting in thrombotic complications [13]. This is clinically significant given that the activation of the coagulation cascade is a common characteristic of DIC and an indication of worse clinical outcome [49].

The D-dimer, a fibrin degradation product, is a relatively small protein fragment produced when plasmin cleaves fibrin to break down clots. The circulating D-dimer concentration may be used as a prognostic biomarker to diagnose thrombotic states, including pulmonary embolism, arterial thrombosis, and DIC [50]. Increased D-dimer was reported in 35–40% of patients with COVID-19, especially in the elderly and those with comorbidities. As a prognostic marker, elevated D-dimer was indicative of disease severity and mortality [51]. The correlation between D-dimer levels and disease severity as well as in-hospital deaths was assessed using the predictive value of D-dimer for in-hospital deaths via receiver operating characteristic analysis [52]. In this analysis, D-dimer was defined as a continuous variable. The findings suggested that an increased D-dimer level on hospital admission (>2.14 mg/L) points to a higher risk for in-hospital deaths [52]. Accordingly, physicians would be better informed about candidates who are more prone to complications and those who would most likely need prompt intervention [52]. The increased D-dimer could be related to DIC or, alternatively, to a sepsis-induced coagulopathy. Even though the exact mechanism for the abnormal thrombotic activity is currently unknown, studies point to the absence of evidence of the existence of DIC in patients with elevated D-dimer who require intensive care unit (ICU) admission due to severe COVID-19 pneumonia [53].

Lymphopenia is caused by severe infections and may be a sign of sepsis. Only a few viral infections may lead to lymphopenia including advanced human immunodeficiency virus (HIV), influenza virus, and viral hepatitis. Other infectious agents that may cause lymphopenia are tuberculosis, typhoid fever, Campylobacter, malaria, and histoplasmosis [54]. Lymphopenia has been related to higher levels of cytokines, especially IL-6 and TNF [15].

Lymphopenia is considered to be an immunological hallmark of COVID-19 infections, but the cause of this association is not entirely clear. It is noteworthy that lymphopenia was described in patients with Middle East respiratory syndrome (MERS), as MERS-CoV can directly infect human primary T lymphocytes and induce intrinsic and extrinsic T-cell apoptosis [55]. Lymphopenia was also reported in patients with SARS-CoV as a result of the involvement of soluble vascular cell adhesion molecule-1 (sVCAM-1), soluble Fas ligand (sFasL), and glucocorticoids [56]. Patients with SARS-CoV experienced intense cytokine storms, which induced apoptosis in lymphocytes and monocytes in a manner similar to what takes place in patients with MERS [56]. In SARS-CoV-2, the underlying mechanisms of reduced lymphocyte counts have not yet been delineated. It was recently suggested that the cause of lymphopenia can be the SARS-CoV-2-induced activation of apoptosis in lymphocytes [57,58]. Alternatively, due to the cytokine storm, lymphocytes may be recruited to the lungs (and other affected organs) leading to their depletion. That might explain why lymphocyte counts are markedly reduced in patients with severe COVID-19 [59]. Lymphocyte counts can also impact the duration of COVID-19. Lower lymphocyte counts have been associated with a longer duration of the symptoms of COVID-19 [60]. Serial measurements of lymphocyte counts can be used as a prognostic marker of patient outcomes [61]. Table 1 summarizes the results of many studies that reported an association between different biomarker levels and severe/poor outcomes in patients with COVID-19. These poor outcomes can lead to severe disease symptoms, higher hospital admission rates, higher rates of mortality, the need for intensive care support, and the need for mechanical ventilation.

## 6. Immune Responses in COVID-19 and Sepsis

Many questions pertinent to the immunopathogenesis of COVID-19 have yet to be answered. These include the following questions and many others. Can someone be re-infected with COVID-19? How long are the COVID-19 antibodies protective for? Can we estimate the disease progression and clinical outcomes of patients with COVID-19 by analyzing their immune profiles shortly after admission? There is currently not enough evidence to answer any of these questions.

Chu et al. [63] compared the effects of SARS-CoV and SARS-CoV-2 on human lung explants and revealed that SARS-CoV-2 had a better capacity to replicate in pulmonary tissues and that both viruses can equally infect type-I and type-II pneumocytes and alveolar macrophages. They demonstrated that SARS-CoV-2 failed to induce IFN-I, IFN-II, and IFN-III, but induced the expression of five other cytokines (IL-6, MCP1, CXCL1, CXCL5, and CXCL10/IP10) [63]. The findings of another study conducted by Blanco-Melo et al. demonstrated that SARS-CoV-2 induced an immune reaction characterized by reduced IFN-I and IFN-III responses and significant induction of various proinflammatory chemokines, IL-1β, IL-6, TNF, and IL1Rα [64]. 

After viruses enter the host cells, they are recognized by pattern recognition receptors (PRRs), which include Toll-like receptors (TLRs) such as TLR7 and TLR8 [64]. Once engaged, these innate immune receptors can induce interferon regulatory factor (IRF), NF-κB, and AP-1, resulting in the production of the Type-I and -III antiviral interferons and other chemokines [65]. These chemokines attract innate cells such as polymorphonuclear leukocytes, NK cells, monocytes, and dendritic cells [66]. These can, in turn, generate MIG, IP-10, MCP-1, and other chemokines that can recruit lymphocytes that are capable of recognizing antigen presentation of the viral antigens [66]. Importantly, the transition between innate and adaptive immune responses appears to be a serious factor for the clinical progression of SARS-CoV-2 infections. Patients with severe COVID-19 show extreme cytokine storms involving the overexpression of IL-2 and IL-6 [67]. Available data indicate a vigorous innate response followed by an inappropriate switch to the adaptive response, which results in immune system exhaustion during the SARS-CoV-2 disease progression [68]. In sepsis, the functional plasticity of monocytes as they switch from a proinflammatory to an immunosuppressive phenotype has been demonstrated [69]. Lymphocyte exhaustion was also reported in sepsis [70]. Thus, T-cell exhaustion is a hallmark that both sepsis and COVID-19 have in common. These effects can be attributed to the activation of immune checkpoints (ICs) and their ligands, such as the programmed death-1 (PD-1) and PD-L1 axis.

The protective response in COVID-19 is T-cell dependent, with the CD4^+^ T helper cells providing help for B cells to secrete their specific neutralizing antibodies, and the cytotoxic CD8^+^ T cells capable of directly clearing viral infected cells. It is noteworthy that 80% of the infiltrating cells in COVID-19 are CD8^+^ T cells [71]. Other key relevant changes in the immune system in the COVID-19 context include lymphocytopenia and a modulation in total neutrophils. Reported data suggest an association between lymphocytopenia and neutrophilia with COVID-19 disease severity and death [72,73]. An evident decline in lymphocytes has been reported [7,29,59]. In addition, there was a reported decrease in the numbers of monocytes, eosinophils, and basophils [73] in patients with COVID-19 who are critically ill (Table 2).

## 7. Final Remarks on COVID-19 and Sepsis

Sepsis is a life-threatening organ dysfunction that results from dysregulated host responses to infection and impaired immune homeostasis [75]. Sepsis has high mortality rates and can be caused by bacteria, fungi, or viruses such as SARS-CoV-2, the causative agent of COVID-19 [75]. The clinical features of sepsis include uncontrolled systemic inflammation that is characterized by a storm of inflammation associated with the release of proinflammatory and anti-inflammatory biomarkers such as IL-6, IL-1, TNF-α, PCT, and CRP [30]. The pathogenesis of sepsis can be explained by the continued activation of neutrophils, macrophages and monocytes due to the inappropriate regulation of immune responses and physiological reactions in response to an infection. In addition, there is delayed apoptosis of neutrophils, and enhanced necrosis of cells and tissues. The resulting dysregulated immune response is a consequence of the close correlation between the coagulation system and the inflammatory response [76]. Severe COVID-19 patients requiring aggressive intensive support frequently present with MOF that involves hypotension and shock, acute respiratory failure, acute kidney injury, and coagulation abnormalities.

There are striking similarities between patients with COVID-19 and those with sepsis. In both cases, cytokines are key players in the hyperinflammation [76]. In both cases, the inflammation leads to the activation of the coagulation cascade, which can cause the activation of the fibrinolytic system [77]. Abnormal coagulation function is a prominent feature in severe COVID-19 cases. This includes increased D-dimer and fibrinogen and the occurrence of venous and arterial thromboembolic events. Possible causes include a direct attack of the virus on the endothelial cells via ACE-2 receptors, cytokine storm, or activation of the coagulation system. Surprisingly, disseminated intravascular coagulation has also been reported in patients with COVID-19 [78]. However, DIC associated with COVID-19 has a different pathophysiology than that of septic DIC. The COVID-19-associated DIC is characterized by an enhanced fibrinolytic system, whereas septic DIC is a DIC with suppressed fibrinolysis [78]. Thus, coagulopathies accompanying severe COVID-19 can cause thromboembolism, but can also go to the other extreme of inducing DIC with a different underlying mechanism than that of sepsis. This might explain the limitations of D-dimer in detecting all causes of COVID-19-associated coagulation abnormalities. Increased coagulation and elevated D-dimer levels were associated with poor prognosis in patients with COVID-19 [51]. Additionally, a substantial proportion of patients who developed sepsis and severe COVID-19 had comorbidities, including diabetes and chronic lung disease [79]. Patients presenting with bacterial sepsis and patients with COVID-19 had similar patterns of MOF. Since SARS-CoV-2 is an infectious pathogen, it is reasonable to conclude that severe COVID-19 is sepsis that is caused by SARS-CoV-2 rather than an uncomplicated inflammatory process [75]. Therapies that prove to be efficacious against COVID-19 should also be successful in treating sepsis through the reduction of the inflammatory response and the anticoagulant effect. Since COVID-19 is a multisystem disease of very high complexity, multiple treatments can be necessary. For example, a combination of remdesivir (to avoid viral replication), cytokine blocking agents (to reduce the inflammatory response), and anticoagulants (to control thrombi formation) can prove to be efficient. 

## 8. Conclusions

Although most patients infected with SARS-CoV-2 develop mild symptoms or are completely asymptomatic, severe cases gain wider attention, especially with the highly contagious ability of this virus [80]. In a manner similar to sepsis, a dysregulated immune response is responsible for the cascade of events occurring in severe COVID-19 cases. This results in a dynamic process that leads to the activation of the adaptive immune system including T lymphocytes and B lymphocytes, which can ultimately lead to cell and tissue necrosis and organ dysfunction. As mentioned in this review, a surge of inflammatory cytokines in sepsis and COVID-19 has been reported. It is of note that ferritin is elevated in both COVID-19 and sepsis. It is evident that hyperferritinemia is a marker of immune dysregulation and uncontrolled inflammation. Although lymphopenia is a common finding in both COVID-19 and sepsis, the immune pathology responsible for COVID-19 lymphopenia is different than that in sepsis. As the coagulation system is linked to the inflammatory process, coagulation abnormalities are manifested in both severe sepsis and critically-ill patients with COVID-19. The coagulation abnormalities accompanying severe COVID-19 manifest a different underlying mechanism than that of sepsis. Thus, it appears that SARS-CoV-2 may possess a unique immune pathology. A more comprehensive understanding of the parameters involved in the development of serious clinical complications (including high disease severity and lethal outcomes) in patients with COVID-19 might improve disease progression, lower the existing burden on health care facilities, and help us identify better therapeutic solutions. Finally, the lessons learned from this pandemic must be implemented in order to make us better prepared for any future attacks by coronaviruses or any other viruses.

## Figures and Tables

**Table 1:** Biomarkers and outcomes in patients hospitalized with COVID-19 (Reference [62]).

Biomarker	Number of Studies	Total Sample Size (Number of Patients)	Biomarker Associated with Higher Risk of Poor Outcomes in COVID-19 Patients
Hematological Biomarkers			
Platelets	17	3481	Thrombocytopenia
Lymphocytes	28	6449	Low lymphocyte count
Inflammatory Biomarkers			
C-reactive Protein (CRP)	20	4843	Elevated CRP
Procalcitonin (PCT)	21	6031	Elevated PCT
Creatine Kinase (CK)	12	1910	Elevated CK
Metabolic Biomarkers			
Aspartate Aminotransferase (AST)	32	6383	Elevated AST
Alanine Aminotransferase (ALT)	13	6019	Elevated ALT
Creatinine	19	3635	Elevated creatinine
Lactate Dehydrogenase (LDH)	18	5394	Elevated LDH
Coagulation Biomarkers			
D-Dimer	16	4862	Elevated D-dimer

**Table 2:** Correlation between immune cells and patients with severe COVID-19 (Reference [74]).

Immune Cells	Number of Studies	Total Sample Size (Number of Patients)	Comments
White Blood Cells	25	4278	Significant increase in white blood cell count in severe COVID-19
Neutrophils	18	2446 (758 severe and 1688 non-severe cases)	Significant increase in neutrophil count in severe COVID-19
T Cells	7	637	T cell responses are critical for the clearance of COVID-19 Delayed T cell response leads to uncontrolled viremia, which drives stronger T cell responses that could aggravate tissue damage
Cytotoxic T Cells	7	637	Functional impairment is observed in severe COVID-19 patients CD8^+^ T cells express more inhibitory receptors in severe cases
Monocytes	7	1128	Activation of blood monocytes was detected in the peripheral blood of patients with severe COVID-19

## Data Availability

Data sharing is not applicable to this article.
